# Supplementary material for: Precision Oncology by Point-of-Care Therapeutic Drug Monitoring and Dosage Adjustment of Conventional Cytotoxic Chemotherapies: A Perspective
Source: Pharmaceutics. 2023 Apr 19;15(4):1283. doi: 10.3390/pharmaceutics15041283 (PMC10147065; doi:10.3390/pharmaceutics15041283)
Supplement: Supplementary file 1 [file pharmaceutics-15-01283-s001.zip › pharmaceutics-2324690-supplementary.pdf]

Table S1. Summary of the exemplified cytotoxic drugs. Possible oncologic indications, common dosage, PK parameters and examples of treatment regimens and co-medications are presented for methotrexate, busulfan, etoposide, 5-fluorouracil, cyclophosphamide and ifosfamide. ALL: acute lymphoblastic leukemia; HSCT: hematopoietic stem cell transplant; AML: acute myeloid leukemia; AUC: area under the curve; C_{ss}: concentration at steady state; i.v.: intravenous; p.o.: *per os*/oral.

Compound	Oncologic indications	Common dosage	Half-life $t_{1/2}$	CL	PK target	Sub-exposure	Over-exposure	Examples of treatment regimens and oncologic co-medications
**METHOTREXATE**	ALL, meningeal leukemia, breast cancer, mycosis fungoides, lung cancer, non-Hodgkin lymphoma	30-50 mg/m² bolus i.v. once per week;<br>15 mg/m² bolus i.v. 5 days every 2-3 weeks<br><br>150 mg/m² bolus i.v. every 2-3 weeks;<br>240 mg/m² i.v. (20min) every 4-7 days;<br>500-1000 mg/m² i.v. (36-48h) every 2-3 weeks<br><br>High dose: 1000-12000 mg/m² i.v. (1-24h) every 1-3 weeks<br>[1]	8-15 h [1]	4.2-21 L/h/m² [2]<br>40-400 mL/min [3]<br>12 L/h [1]	Mean peak serum threshold at 1000 µmol/L (< 1500 µmol/L) after 6-hour infusion in high-dose regimen [4]<br><br>Serum level maintained at 700 µmol/L [5]<br><br>C_{ss} 16–40 µmol/L for 24-hour infusion in children with ALL [6]	Treatment non-efficacy	Acute kidney injury, myelosupression, mucositis, dermatological side effects, hepatotoxicity [7]	Leucovorin (folinic acid)<br>5-Fluorouracil<br>Cyclophosphamide<br>Vincristine<br>MIED : methotrexate, ifosfamide, etoposide and dexamethasone [8]
**BUSULFAN**	Conditioning regimen prior to HSCT	2-hour i.v. infusion of 0.8mg/kg every 6 hours for 4 days [9]	Adult: 2.8-3.9 h [9]<br>Pediatric: 2.24-2.5 h [9]	0.22 L/h/kg [10]<br>Adult: 2.25-2.74 ml/min/kg [9]<br>Pediatric: 2.52-3.97 ml/min/kg [9]	AUC_{cum} 78-101 mg·h/L [11]	Graft failure, relapse [11]	Veno-occlusive disease, graft-versus-host disease, mucositis, transplantation-related mortality [11]	BuCy/CyBu: busulfan, cyclophosphamide [12]<br>Fludarabine<br>Thiotepa<br>Melphalan

**ETOPOSIDE**	Hodgkin and non-Hodgkin lymphoma, testicular cancer, lung cancer, gastrointestinal cancer, breast cancer, pre-HSCT regimen, hepatocellular carcinoma	50-100 mg/m² i.v. on days 1-5 or 100 mg/m² i.v. on days 1, 3 and 5 of a 3-4 weeks treatment cycle [13]	Terminal: 3-12 h [13]	18.5 mL/min [14]	AUC 4.6-8.2 mg·min/mL [15]	Treatment non-efficacy	Myelosupression [16], palmar erhythema [17]	BEAM: carmustine, etoposide, cytarabine, melphalan [18]<br>ICE: ifosfamide, carboplatin, etoposide [19]<br>EPOCH: etoposide, cyclophosphamide, doxorubicin, vincristine, prednisone [20]<br>AVI: doxorubicin, etoposide, ifosfamide [21]<br>Cisplatin<br>Bleomycin<br>MIED : methotrexate, ifosfamide, etoposide and dexamethasone [8]
**5-FLUOROURACIL**	Colorectal cancer, head and neck cancer, breast cancer, gastrointestinal cancers, skin cancer (incl. pre-cancerous lesions, topical application)	300-600 mg/m² slow bolus or 24h i.v. (< 1g/day) [22]	Dose-dependent, 5-20 min [22]	5 L/min [4]	AUC 20-25 mg·h/L [23]<br>AUC 20-30 mg·h/ L [24]	Treatment non-efficacy	Diarrhea, vomiting, nausea, neutropenia, pyrexia, pulmonary embolism, thrombocytopenia, leukopenia [25]	LV5FU: leucovorin, 5-FU [26]<br>FOLFOX: leucovorin, 5-FU, oxaliplatin [26]<br>FOLFIRI: leucovorin, 5-FU, irinotecan hydrochloride [26]<br>Bevacizumab<br>Cisplatin<br>Mitomycin-C<br>Streptozocin<br>Doxorubicin<br>Carboplatin<br>Methotrexate<br>Cyclophosphamide
**CYCLOPHOSPHAMIDE**	Conditioning regimen prior to HSCT, ALL, AML, Hodgkin and non-Hodgkin lymphoma, plasmocytoma, ovarian carcinoma, seminoma,	Continuous treatment: 3-6 mg/kg i.v. daily 50-200 mg/day p.o.;<br><br>Intermittent treatment: 10-15 mg/kg i.v. at 2–5 days interval;<br><br>High-dose intermittent	6-9 h [27]	< 2 years old: 46.6 mL/min/m² [28]<br>Renal CL: 5-11 mL/min [27]	Hydroxy-CPA: AUC >50 µmol/L<br>Carboxyethylphosphoramide mustard: AUC 325±25 µmol/L [29]	Treatment non-efficacy	Urinary tract toxicity, hemorrhagic cystitis, cardiotoxicity, pulmonary toxicity, hepatic veno-occlusive disease [30]	BuCy/CyBu: busulfan, cyclophosphamide [12]<br>CHOP: cyclophosphamide, doxorubicin, vincristine, prednisone (R-CHOP: + rituximab [31] ; EPOCH: + etoposide [20]) [32]<br>CTC: cyclophosphamide, thiotepa, carboplatin [33]<br>5-Fluorouracil<br>Methotrexate<br>Epirubicin

	breast carcinoma, small cell bronchogenic carcinoma, neuroblastoma, Ewing's sarcoma	treatment: 20-40 mg/kg i.v. every 21 to 28 days [27]						Mesna
**IFOSFAMIDE**	Hodgkin and non-Hodgkin lymphoma, soft tissue sarcomas, Ewing sarcoma, cervical cancer, lung carcinoma, osteosarcoma, thymic cancer, ovarian cancer	30-60 mg/kg i.v. (0.5-2h) per day for 5 days;<br><br>125-200 mg/kg continuous i.v. over 24h [34]	4-7 h [34]	3.6-8.02 L/h depending on the posology [35]<br>Renal CL: 6-22 mL/min [34]	NA	Treatment non-efficacy	Nausea, vomiting, encephalopathy, hemorrhagic cystitis, metabolic acidosis, hepatic necrosis (result of hepatic veno-occlusive disease) [36], Fanconi syndrome [37]	ICE: ifosfamide, carboplatin, etoposide [19]<br>AVI: doxorubicin, etoposide, ifosfamide [38]<br>MAID: doxorubicin, ifosfamide, mesna, dacarbazine [39]<br>AIM: doxorubicin, ifosfamide, mesna [40]<br>MIED : methotrexate, ifosfamide, etoposide and dexamethasone [8]<br>Mesna

1. SwissMedicInfo, Méthotrexate Sandoz® solution à diluer pour solution injectable/pour perfusion, Sandoz Pharmaceuticals AG. Available online: https://swissmedicinfo.ch/showText.aspx?textType=FI&lang=FR&authNr=51079 (accessed on 22.02.2023).
2. Dao, K.; Ivanyuk, A.; Buclin, T.; Beck-Popovic, M.; Diezi, M. Pharmacokinetic interaction between methotrexate and chloral hydrate. *Pediatr Blood Cancer* **2013**, *60*, 518-520, doi:10.1002/pbc.24393.
3. Leveque, D.; Santucci, R.; Gourieux, B.; Herbrecht, R. Pharmacokinetic drug-drug interactions with methotrexate in oncology. *Expert Rev Clin Pharmacol* **2011**, *4*, 743-750, doi:10.1586/ecp.11.57.

4. Paci, A.; Veal, G.; Bardin, C.; Leveque, D.; Widmer, N.; Beijnen, J.; Astier, A.; Chatelut, E. Review of therapeutic drug monitoring of anticancer drugs part 1--cytotoxics. *Eur J Cancer* **2014**, *50*, 2010-2019, doi:10.1016/j.ejca.2014.04.014.
5. Fujita, Y.; Nakamura, T.; Aomori, T.; Nishiba, H.; Shinozaki, T.; Yanagawa, T.; Takagishi, K.; Watanabe, H.; Okada, Y.; Nakamura, K.; et al. Pharmacokinetic Individualization of High-Dose Methotrexate Chemotherapy for the Treatment of Localized Osteosarcoma. *J Chemotherapy* **2010**, *22*, 186-190, doi:DOI 10.1179/joc.2010.22.3.186.
6. Song, Z.; Hu, Y.; Liu, S.; Wang, G.; Zhai, S.; Zhang, X.; Li, Y.; Du, G.; Shi, Y.; Chen, Y.; et al. Medication therapy of high-dose methotrexate: An evidence-based practice guideline of the Division of Therapeutic Drug Monitoring, Chinese Pharmacological Society. *Br J Clin Pharmacol* **2022**, *88*, 2456-2472, doi:10.1111/bcp.15134.
7. Howard, S.C.; McCormick, J.; Pui, C.H.; Buddington, R.K.; Harvey, R.D. Preventing and Managing Toxicities of High-Dose Methotrexate. *Oncologist* **2016**, *21*, 1471-1482, doi:10.1634/theoncologist.2015-0164.
8. Sandlund, J.T.; Pui, C.H.; Mahmoud, H.; Zhou, Y.; Lowe, E.; Kaste, S.; Kun, L.E.; Krasin, M.J.; Onciu, M.; Behm, F.G.; et al. Efficacy of high-dose methotrexate, ifosfamide, etoposide and dexamethasone salvage therapy for recurrent or refractory childhood malignant lymphoma. *Ann Oncol* **2011**, *22*, 468-471, doi:10.1093/annonc/mdq348.
9. SwissMedicInfo, Busilvex®, Pierre Fabre Pharma AG. Available online: https://swissmedicinfo.ch/showText.aspx?textType=FI&lang=FR&authNr=56739 (accessed on 22.02.2023).
10. Lawson, R.; Staatz, C.E.; Fraser, C.J.; Hennig, S. Review of the Pharmacokinetics and Pharmacodynamics of Intravenous Busulfan in Paediatric Patients. *Clin Pharmacokinet* **2021**, *60*, 17-51, doi:10.1007/s40262-020-00947-2.
11. Bartelink, I.H.; Lalmohamed, A.; van Reij, E.M.; Dvorak, C.C.; Savic, R.M.; Zwaveling, J.; Bredius, R.G.; Egberts, A.C.; Bierings, M.; Kletzel, M.; et al. Association of busulfan exposure with survival and toxicity after haemopoietic cell transplantation in children and young adults: a multicentre, retrospective cohort analysis. *Lancet Haematol* **2016**, *3*, e526-e536, doi:10.1016/S2352-3026(16)30114-4.
12. Seydoux, C.; Medinger, M.; Gerull, S.; Halter, J.; Heim, D.; Chalandon, Y.; Levrat, S.M.; Schanz, U.; Nair, G.; Ansari, M.; et al. Busulfan-cyclophosphamide versus cyclophosphamide-busulfan as conditioning regimen before allogeneic hematopoietic cell transplantation: a prospective randomized trial. *Ann Hematol* **2021**, *100*, 209-216, doi:10.1007/s00277-020-04312-y.
13. SwissMedicInfo, Etoposide Sandoz®, Sandoz Pharmaceuticals AG. Available online: https://swissmedicinfo.ch/showText.aspx?textType=FI&lang=FR&authNr=55165 (accessed on 22.02.2023).
14. Kroschinsky, F.P.; Friedrichsen, K.; Mueller, J.; Pursche, S.; Haenel, M.; Prondzinsky, R.; Ehninger, G.; Schleyer, E. Pharmacokinetic comparison of oral and intravenous etoposide in patients treated with the CHOEP-regimen for malignant lymphomas. *Cancer Chemother Pharmacol* **2008**, *61*, 785-790, doi:10.1007/s00280-007-0535-3.
15. Lowis, S.P.; Price, L.; Pearson, A.D.; Newell, D.R.; Cole, M. A study of the feasibility and accuracy of pharmacokinetically guided etoposide dosing in children. *Br J Cancer* **1998**, *77*, 2318-2323, doi:10.1038/bjc.1998.385.
16. Reyhanoglu, G.; Tadi, P. Etoposide. In *StatPearls*; Treasure Island (FL), 2022.
17. Murphy, C.P.; Harden, E.A.; Herzig, R.H. Dose-related cutaneous toxicities with etoposide. *Cancer* **1993**, *71*, 3153-3155, doi:10.1002/1097-0142(19930515)71:10<3153::aid-cncr2820711042>3.0.co;2-#.

18. Gunes, A.K.; Serin, I.; Demir, I.; Sarifakiogullari, S.; Durusoy, S.S.; Akkurt, D.M.; Ince, I.; Ozkan, G.; Cinli, T.A.; Pehlivan, M. Comparison of Mitoxantrone-Melphalan and BEAM Conditioning Regimens in Patients with Lymphoma. *Hematol Oncol Stem Cell Ther* **2022**, *15*, 201-207, doi:10.1016/j.hemonc.2021.03.005.
19. Zelenetz, A.D.; Hamlin, P.; Kewalramani, T.; Yahalom, J.; Nimer, S.; Moskowitz, C.H. Ifosfamide, carboplatin, etoposide (ICE)-based second-line chemotherapy for the management of relapsed and refractory aggressive non-Hodgkin's lymphoma. *Ann Oncol* **2003**, *14 Suppl 1*, i5-10, doi:10.1093/annonc/mdg702.
20. Wilson, W.H.; Bryant, G.; Bates, S.; Fojo, A.; Wittes, R.E.; Steinberg, S.M.; Kohler, D.R.; Jaffe, E.S.; Herdt, J.; Cheson, B.D.; et al. EPOCH chemotherapy: toxicity and efficacy in relapsed and refractory non-Hodgkin's lymphoma. *J Clin Oncol* **1993**, *11*, 1573-1582, doi:10.1200/JCO.1993.11.8.1573.
21. Trillet-Lenoir, V.; Mornex, F.; Chauvin, F.; Fournel, P.; Voloch, A.; Perol, M.; Laennec, E.; Piperno, D.; Boyer, J.; Ardiet, J.M.; et al. Limited disease small cell lung cancer: alternating combination of doxorubicin, etoposide, ifosfamide and hyperfractionated radiotherapy. Final results of a multicentric pilot study for the Groupe Lyonnias d'Oncologie Thoracique (GLOT). *Lung Cancer* **1993**, *10*, 35-45, doi:10.1016/0169-5002(93)90307-j.
22. SwissMedicInfo, Fluorouracile Sandoz®, Sandoz Pharmaceuticals AG. Available online: https://swissmedicinfo.ch/showText.aspx?textType=FI&lang=FR&authNr=60186 (accessed on 22.02.2023).
23. Saif, M.W.; Choma, A.; Salamone, S.J.; Chu, E. Pharmacokinetically guided dose adjustment of 5-fluorouracil: a rational approach to improving therapeutic outcomes. *J Natl Cancer Inst* **2009**, *101*, 1543-1552, doi:10.1093/jnci/djp328.
24. Kaldate, R.R.; Haregewoin, A.; Grier, C.E.; Hamilton, S.A.; McLeod, H.L. Modeling the 5-fluorouracil area under the curve versus dose relationship to develop a pharmacokinetic dosing algorithm for colorectal cancer patients receiving FOLFOX6. *Oncologist* **2012**, *17*, 296-302, doi:10.1634/theoncologist.2011-0357.
25. Casale, J.; Patel, P. Fluorouracil. In *StatPearls*; Treasure Island (FL), 2022.
26. Mabro, M.; Artru, P.; Andre, T.; Flesch, M.; Maindrault-Goebel, F.; Landi, B.; Lledo, G.; Plantade, A.; Louvet, C.; de Gramont, A. A phase II study of FOLFIRI-3 (double infusion of irinotecan combined with LV5FU) after FOLFOX in advanced colorectal cancer patients. *Br J Cancer* **2006**, *94*, 1287-1292, doi:10.1038/sj.bjc.6603095.
27. SwissMedicInfo, Endoxan, Baxter AG. Available online: https://swissmedicinfo.ch/showText.aspx?textType=FI&lang=FR&authNr=25442 (accessed on 22.02.2023).
28. Barnett, S.; Errington, J.; Sludden, J.; Jamieson, D.; Poinsignon, V.; Paci, A.; Veal, G.J. Pharmacokinetics and Pharmacogenetics of Cyclophosphamide in a Neonate and Infant Childhood Cancer Patient Population. *Pharmaceuticals (Basel)* **2021**, *14*, doi:10.3390/ph14030272.
29. Salinger, D.H.; McCune, J.S.; Ren, A.G.; Shen, D.D.; Slattery, J.T.; Phillips, B.; McDonald, G.B.; Vicini, P. Real-time dose adjustment of cyclophosphamide in a preparative regimen for hematopoietic cell transplant: a Bayesian pharmacokinetic approach. *Clin Cancer Res* **2006**, *12*, 4888-4898, doi:10.1158/1078-0432.CCR-05-2079.
30. Ogino, M.H.; Tadi, P. Cyclophosphamide. In *StatPearls*; Treasure Island (FL), 2022.
31. Miura, K.; Takahashi, H.; Nakagawa, M.; Hamada, T.; Uchino, Y.; Iizuka, K.; Ohtake, S.; Iriyama, N.; Hatta, Y.; Nakamura, H. Ideal dose intensity of R-CHOP in diffuse large B-cell lymphoma. *Expert Rev Anticancer Ther* **2022**, *22*, 583-595, doi:10.1080/14737140.2022.2071262.

32. Fisher, R.I.; Gaynor, E.R.; Dahlberg, S.; Oken, M.M.; Grogan, T.M.; Mize, E.M.; Glick, J.H.; Coltman, C.A., Jr.; Miller, T.P. Comparison of a standard regimen (CHOP) with three intensive chemotherapy regimens for advanced non-Hodgkin's lymphoma. *N Engl J Med* **1993**, *328*, 1002-1006, doi:10.1056/NEJM199304083281404.
33. Schrama, J.G.; Holtkamp, M.J.; Baars, J.W.; Schornagel, J.H.; Rodenhuis, S. Toxicity of the high-dose chemotherapy CTC regimen (cyclophosphamide, thiotepa, carboplatin): the Netherlands Cancer Institute experience. *Br J Cancer* **2003**, *88*, 1831-1838, doi:10.1038/sj.bjc.6601001.
34. SwissMedicInfo, Holoxan, Baxter AG. Available online: https://swissmedicinfo.ch/showText.aspx?textType=FI&lang=FR&authNr=41217 (accessed on 22.02.2023).
35. Kerbusch, T.; de Kraker, J.; Keizer, H.J.; van Putten, J.W.; Groen, H.J.; Jansen, R.L.; Schellens, J.H.; Beijnen, J.H. Clinical pharmacokinetics and pharmacodynamics of ifosfamide and its metabolites. *Clin Pharmacokinet* **2001**, *40*, 41-62, doi:10.2165/00003088-200140010-00004.
36. Gangireddy, M.; Nookala, V. Ifosfamide. In *StatPearls*; Treasure Island (FL), 2022.
37. Buttemer, S.; Pai, M.; Lau, K.K. Ifosfamide induced Fanconi syndrome. *BMJ Case Rep* **2011**, *2011*, doi:10.1136/bcr.10.2011.4950.
38. Freyer, G.; Ligneau, B.; Tranchand, B.; Ardiet, C.; Souquet, P.J.; Court-Fortune, I.; Riou, R.; Rebattu, P.; Morignat, E.; Boissel, J.P.; et al. The prognostic value of etoposide area under the curve (AUC) at first chemotherapy cycle in small cell lung cancer patients: a multicenter study of the groupe Lyon-Saint-Etienne d'Oncologie Thoracique (GLOT). *Lung Cancer* **2001**, *31*, 247-256, doi:10.1016/s0169-5002(00)00174-4.
39. Pearl, M.L.; Inagami, M.; McCauley, D.L.; Valea, F.A.; Chalas, E.; Fischer, M. Mesna, doxorubicin, ifosfamide, and dacarbazine (MAID) chemotherapy for gynecological sarcomas. *Int J Gynecol Cancer* **2002**, *12*, 745-748, doi:10.1046/j.1525-1438.2002.01139.x.
40. Steen, S.; Stephenson, G. Current treatment of soft tissue sarcoma. *Proc (Bayl Univ Med Cent)* **2008**, *21*, 392-396, doi:10.1080/08998280.2008.11928435.
